# Role of Social Media in the Rising Body Dissatisfaction and Dysmorphia Among Adolescents

**DOI:** 10.7759/cureus.78314

**Published:** 2025-01-31

**Authors:** Aakriti Khajuria, Anandhi Gandotra, Anmol Khajuria, Kritika Arora, Ravinder K Gupta, Urvi Gupta, Sunny Babber

**Affiliations:** 1 Department of Psychiatry, Acharya Shri Chander College of Medical Sciences and Hospital, Jammu, IND; 2 Department of Community Medicine, Acharya Shri Chander College of Medical Sciences and Hospital, Jammu, IND; 3 Department of Pediatrics, Acharya Shri Chander College of Medical Sciences and Hospital, Jammu, IND; 4 Clinical Psychology, Sharda University, Delhi, IND

**Keywords:** adolescents, body dysmorphic disorder (bdd), dysmorphic concern questionnaire (dcq), influencers, selfies, social media

## Abstract

Background

The growing use of social media has a major impact on people’s lives, especially adolescents. Teenagers tend to upload pictures, reels, and stories to keep up with the latest trends, seek approval from society, and gain self-worth. Influencers' heavy use of photoshopped, edited images using filters is setting certain unreal beauty standards, and adolescents trying to copy them is leading to an increase in body dysmorphic disorder (BDD).

Aim and objective

This study aimed to study the prevalence of BDD in adolescents and its association with social media usage.

Material and methods

A descriptive cross-sectional, validated questionnaire-based study was conducted among adolescents in the age group of 13-18 years by the Department of Pediatrics of Acharya Shri Chander College of Medical Science and Hospital (ASCOMS & H) in Jammu, India. Data were collected by distributing the validated questionnaire consisting of sociodemographic data, social media use, and the Dysmorphic Concern Questionnaire (DCQ) scale from four schools (two government and two private) in Jammu. A total of 740 completely filled questionnaires were analyzed statistically. Analysis of variance (ANOVA) was used to compare the mean scores of BDD, whereas the Chi-square and Kruskal-Wallis test for non-parametric data and p < 0.05 was considered statistically significant.

Results

In the present study, the prevalence of BDD in adolescents in Jammu is found to be 29.45%. Greater frequency of social media usage, i.e., greater than four hours a day, social media practices like taking more selfies, excess use of beauty filters, and sharing of pictures on a regular basis, were related to increased mean DCQ scores and thus an increase in BDD. The study showed that with the increase in age, there was a subsequent increase in the prevalence of BDD. Female gender and adolescents having a lower economic status at home significantly have more chance to develop BDD.

Conclusion

Our study concludes that there is a potential association between the use of social media in rising BDD, especially among females and adolescents of lower economic status. Doctors, school teachers, parents, friends, government campaigns, policymakers, and social media influencers can come together to spread awareness and educate adolescents about self-love, confidence, and natural beauty.

## Introduction

Social media refers to the various online platforms that have become a means of interaction, communication, sharing views, and exchange of data with others. It allows people to connect with others, spread awareness, elaborate business, and help in studies, offering opportunities for networking and entertainment. In this era of social media, everyone has a social media account, and it has become as important as having an address or email ID. People not using social media are considered regressive and out of date. After the COVID-19 pandemic, studies also shifted to online mode; therefore, it is nearly impossible to keep adolescents away from the internet and social media [1]. Although people of all ages use social media, its usage has specifically increased among adolescents. In India, adolescents between the age group of 10 years to 19 years make up 31% of the overall number of people who use social media [2].

Adolescence is the phase that helps with the transition of an individual into an adult, making it a very vulnerable phase and the content on social media being accessed by the teenager impacts his/her mental health and self-esteem [3]. Body image refers to a person's emotional attitudes, beliefs, and perceptions of their own body that is what a person believes about their appearance, for example, how they feel about their body, height, weight, and shape [4]. Positive body image means body satisfaction and acceptance, whereas negative body image is related to dissatisfaction and wanting one’s body to be different. A negative body image can sometimes contribute to mental health issues like depression and anxiety, eating disorders like anorexia nervosa, and body dysmorphic disorder (BDD). Teenagers having BDD tend to perform repetitive acts such as checking mirrors, weighing themselves, or comparing their appearance with others, and this takes a toll on their mental health, and performance at work and school also decreases. The affected adolescents might also lose interest in school activities and avoid any kind of social interaction.

Users of social media platforms who have followers in thousands and millions are nowadays recognized as celebrities and they not only promote their businesses and ideologies but also collaborate with brands to promote their products. Seeing their popularity, everyone wishes to go "viral’’ and become the so-called “influencers," Instagram models, and Twitter and Facebook celebrities [5]. These influencers and celebrities post photos with excellent editing, bright colors, and various filters that are unreal, but the teenagers following them set them as an ideal to be achieved. Adolescents, while scrolling through feeds and timelines on Instagram, get exposed to these unreal beauty standards as every other reel that they see has these influencers selling some beauty product to achieve a certain kind of look, color, or shape. Eventually, they start believing in themselves, start feeling bad about their bodies, and try to achieve unrealistic body structure and appearance, which results in BDD.

Reels with engaging captions like "five ways to get slimmer," "seven ways to grow your hair," "getting one tone fairer within three days," and so on make teenagers believe that they are lacking in something and they need to achieve that. These advertisements and reels send an underlying message to teenagers about how they should dress and how they should maintain their physical attractiveness and health. According to research by Khunger and Pant [6], teenagers between 13 and 19 years are increasingly seeking cosmetic procedures and were found to be suffering from depression, anxiety, and low self-esteem due to obsession with body image and celebrity culture fueled by social networking sites. A study done by Panjrath and Tiwari [7] highlights how these social media influencers by showcasing their perfect hourglass-shaped bodies on social media have led to an increase in body dissatisfaction, especially in adolescent girls.

Social media use and behavior can be determined using pre-validated questionnaires in various studies [8], which use questions to access the activities done by individuals pertaining to social media. The presence or absence of BDD in an individual can be accessed by using mainly the Dysmorphic Concern Questionnaire (DCQ) scale, which is composed of seven questions with a four-point scale used for screening of BDD (e.g., mirror checking, skin picking, excessive grooming, comparing with others, feeling the need for surgery, and worrying about defect) [9]. Keeping this in mind, we tried to assess the relationship between social media consumption and BDD among adolescents. Findings from this study can help policymakers in formulating better regulations and practices.

## Materials and methods

Study design

This research was designed as a descriptive cross-sectional and questionnaires-based study among Indian adolescents after getting approval from the Institutional Independent Ethical Committee with reference no. ASCOMS/IEC/2024/Meeting-II/FM/23 dated 18/05/24, in the Post-graduate Department of Pediatrics from June 1, 2024, to August 31, 2024, at Acharya Shri Chander College of Medical Sciences and Hospital (ASCOMS & H) in Jammu, India, over a period of three months. Several schools of the Jammu region (government and private) were approached with information about the study and were invited to take part via phone and email. Four schools (two government and two private) responded and were considered appropriate as they all were co-educational and represented different regions of Jammu. Students from the age group 13-17 years were considered for our study. The pre-validated questionnaire form was distributed to a total of 860 students during regular visits to the schools (see Appendix).

After three months, 120 forms were found to be incomplete, and therefore we were left with 740 completely filled questionnaire forms, which were converted to data on a Microsoft Excel sheet (Microsoft Corporation, Redmond, WA, USA) for further analysis. The questionnaire was divided into three parts: (I) sociodemographic data, (II) social media use and behaviors, and (III) the DCQ scale. Social media use and behaviors were assessed via a four-point Likert scale (all of the time, sometimes, rarely, and never) [9]. The DCQ is a self-reporting questionnaire consisting of seven questions that access dysmorphic concern and evaluated on a four-point Likert scale with scoring (0 score for “never,” 1 for “rarely," 2 for “sometimes,” and 3 for “all of the time”). As we had seven questions, this scale could vary from 0 to 21 where higher scores indicate an increasing level of body dysmorphia and vice versa; a cut-off score of 11 or more was selected to identify participants having BDD as this cut-off showed good internal consistency with Cronbach’s α = 0.81 [9]. 

Inclusive Criteria

All adolescents in the age group of 13-18 years of age who are available in the schools and are capable of filling out the questionnaires were included in our study.

Exclusive Criteria

Adolescents less than 13 years and greater than 18 years were excluded from our study because adolescents less than 13 are not able to completely understand the given questionnaire and adolescents greater than 18 years are difficult to find in the schools.

Statistical analysis

A statistical analysis was done by using IBM SPSS Statistics for Windows, Version 21.0 (released 2012, IBM Corp., Armonk, NY). Descriptive data were expressed in terms of percentages and compared by using the Chi-square test and Kruskal-Wallis test, whereas continuous data were expressed in terms of mean and standard deviation and compared using the analysis of variance (ANOVA) test. A p-value less than 0.05 was considered as statistically significant, otherwise nonsignificant.

## Results

A total of 740 completely filled questionnaires were statistically analyzed. Table 1 shows the sociodemographic profile of the study population. Out of 740, 190 respondents were in the age group of 13-14 years (25.68%), 256 participants were aged 15-16 years old (34.59 %), while 294 were in ages of 17-18 years old (39.73%). Among the respondents, 393 (53.11%) were males, and 347 (46.89%) were females. Economically, the highest percentage of students admitted that their parents had a lower-middle-class status (492, 66.49%), followed by 132 (17.84%) students whose family had a middle-class status, succeeded by an upper-class status as per 78 (10.54%) participants, while 48 (6.49%) students said that their family earnings lead to a low-class status.

**Table 1:** Demographics of the respondents

Demographical profile		No. of respondents	Percentage (%)
Age (in years)	13-14	190	25.68
	15-16	256	34.59
	17-18	294	39.73
Gender	Male	393	53.11
	Female	347	46.89
Economic status	Lower class	48	6.49
	Lower-middle class	492	66.49
	Middle class	132	17.84
	Upper class	78	10.54

Table 2 shows the social media practices of the respondents where the maximum number of students, i.e., 396 (53.51%), spent one to three hours a day on Instagram, Facebook, and Snapchat apps, while 138 (18.65%) said that they used these social media sites for less than one hour per day only. About 114 (15.41%) spent four to seven hours daily on social media, whereas 92 students (12.43%) admitted that they spend more than seven hours daily. It also shows that 75 students (10.14%) never took selfies while a maximum of students (289, 39.05%) rarely took selfies. About 263 (35.54%) took selfies sometimes, and 113 students (15.27%) admitted that they always took selfies. Most of the students, i.e., 312 (42.16%), said that they tend to use filters to edit selfies sometimes, whereas 208 (28.11%) say that they rarely used filters to edit selfies. About 136 (18.38%) of participants stick to the fact that they never use filters and 84 (11.35%) of them admitted that they always use filters to edit selfies. Lastly, Table 2 gives an insight into the sharing of selfies with others, where most of the respondents (28, 38.65%) say that they sometimes share their selfies with others and 252 (34.05%) confirmed that they rarely do so. A total of 106 (14.32%) deny sharing selfies and 96 (12.97%) say that they always do so.

**Table 2:** Social media practices of the respondents

Social media practices		No. of respondents	Percentage (%)
Time spent on Instagram/Snapchat/Facebook etc. per day	<1 hour	138	18.65
	1-3 hours	396	53.51
	4-7 hours	114	15.41
	>7 hours	92	12.43
Take selfies	Never	75	10.14
	Rarely	289	39.05
	Sometimes	263	35.54
	Always	113	15.27
Use filters to edit selfies	Never	136	18.38
	Rarely	208	28.11
	Sometimes	312	42.16
	Always	84	11.35
Share selfies with others	Never	106	14.32
	Rarely	252	34.05
	Sometimes	286	38.65
	Always	96	12.97

According to Table 3, the mean DCQ scores related to social media practices show respondents who spent four to seven hours or greater than seven hours a day on social media had significantly high DCQ scores than those who spent less than one hour or one to three hours per day (p = 0.0001). Students who always and sometimes took selfies had more dysmorphic concerns as compared to the ones who rarely or never took selfies (p = 0.0001). Lastly, Table 3 shows that the students who always and sometimes shared selfies with others tend to have more dysmorphic scores in contrast to the students who rarely or never did so (p = 0.0021).

**Table 3:** Mean Dysmorphic Concern Questionnaire (DCQ) scores related to social media practices of the respondents. Analysis of variance (ANOVA) test and Kruskal Wallis test were used for the analysis. * indicates statistical significance.

Social media practices		Mean (M)	Standard deviation (SD)	p-value
Time spent on Instagram/Snapchat/Twitter (X)/Facebook etc. per day	<1 hour	5.12	4.32	0.0001*
	1-3 hours	5.78	4.86	
	4-7 hours	7.43	6.13	
	>7 hours	7.96	7.19	
Take selfies	Never	5.18	4.67	0.0001*
	Rarely	6.32	5.14	
	Sometimes	7.46	6.12	
	Always	7.89	5.86	
Use filters to edit selfies	Never	4.28	3.13	0.0001*
	Rarely	4.87	3.18	
	Sometimes	5.72	4.16	
	Always	8.42	5.43	
Share selfies with others	Never	6.52	4.14	0.0021*
	Rarely	6.86	4.51	
	Sometimes	7.12	3.96	
	Always	7.48	3.98	

Table 4 highlights the association of BDD (DCQ score of 11 and higher) with sociodemographic characteristics of the study population. The prevalence of BDD in our study population is 29.45%, and the prevalence increased with the subsequent increase in age with the highest cases (35.92%) in the 17-18-year age group, followed by 27.74% cases in the 15-16-year age group and 22.5% cases in the 13-14-year age group (p = 0.0005). About 26.21% of males had BDD while 33.14% of females were affected, which shows that the female gender is more likely to develop BDD as compared to males (p = 0.0389). The above table also highlights the fact that the adolescents in the lower class have more inclination towards the development of BDD (43.40%) as compared to the ones in the middle-class (23.95%) and upper-class families (28.21%) (p = 0.0068).

**Table 4:** Comparison of prevalence of body dysmorphic disorder (BDD) by demographic characteristics. The chi-square test is used for the analysis of demographical data. * indicates statistical significance. DCQ: Dysmorphic Concern Questionnaire

Demographical profile		Normal (DCQ < 11)		BDD (DCQ ≥ 11)		p-value
		N = 522	%	N = 218	%	
Age (in years)	13-14	155	77.5	45	22.5	0.0005*
	15-16	185	72.26	71	27.74	
	17-18	182	64.08	102	35.92	
Gender	Male	290	73.79	103	26.21	0.0389*
	Female	232	66.86	115	33.14	
Economic status	Lower class	30	56.60	23	43.40	0.0068*
	Lower-middle class	328	70.23	139	29.77	
	Middle class	108	76.05	34	23.95	
	Upper class	56	71.79	22	28.21	

## Discussion

There is a growing prevalence of BDD in adolescents, and the present cross-sectional study seeks to contribute to exploring the association between the use of social media by adolescents and body dysmorphic symptoms. In our study, greater frequency of social media usage (Instagram/Snapchat/Twitter (X)/Facebook) for more than four hours a day was related to increased mean DCQ scores. This finding is in line with the existing literature by Gupta et al. [10] conducted on adolescents 16-18 years of age, which also highlights that the association was more specific to social media platforms, which are highly image-based (like Instagram and Youtube Shorts), as opposed to text-based platforms (like X formerly known as Twitter). Our study also shows that adolescents who take more selfies and frequently share these photos have more dysmorphic concerns as compared to students who never or rarely take selfies and share them. The same has been proven in a previous study [11], which shows the link between social media use and behavior, particularly taking frequent “selfies” and posting them as stories and statuses on Instagram handles and WhatsApp accounts, which leads to increased body dissatisfaction and over-evaluation of own looks, shape, and weight by an individual, especially in young adults. The current study highlights the fact that adolescents who always used filters to edit selfies were more likely to develop dysmorphic concern when compared to the ones who rarely or never used filters to edit their pictures. A study conducted by McLean et al. [12] shows that adolescents who regularly photoshopped their selfies and used photo-editing tools before posting their pictures online are associated with body dissatisfaction and increased concerns for their overall looks. Another research done in California [13] suggests that social media platforms like Snapchat offer a beauty filter, which is a photo-editing tool that allows users to smooth out their skin, contour their nose, enhance their lips and eyes, alter their jawline and cheekbones, etc. These beauty filters make young women form a fixed image of beauty standards and how they should look, and this has not only led to rising body dissatisfaction but also these adolescents are now seeking cosmetic surgeons to alter their appearance to look just like their filtered photos (this trend is referred to as Snapchat dysmorphia [13]).

In the present study, the prevalence of BDD in adolescents of Jammu (a city in northern India) is found to be 29.45%. Similarly, research conducted in Jharkhand (central India) [14] showed that 34.3% of adolescents had significant overall appearance concerns, while 20.5% had concerns about shape or body build. By contrast, in a study in Goa (southern India) [15], only 6.9% were found to have been diagnosed with BDD although 45.8% of the total students surveyed experienced slight distress due to their physical features. The disparity between the prevalence rates of BDD could be attributed to differences in the sample size and the lack of a unified screening method. Our study showed that with the increase in age from 13 to 17 years, there was a subsequent increase in the prevalence of BDD. A similar trend was seen in research by Bhavya Narang in Uttar Pradesh, India [16], and by Himanshu et al. in Punjab, India [17]. We also observed that females are more prone to develop BDD as compared to males. This is in sync with almost all the previous literature. The above study of Punjab [17] shows the female participants of their study to be more dissatisfied with their bodies in comparison to their male counterparts. It emphasizes that in females, areas of concern are mainly hirsutism, fat, color complexion, height, etc., whereas in males, the areas of concern are generally their height, muscular build, acne, weight, hair thinning, and premature greying. Another study conducted in Kolkata [18] highlights that females not only are more prone to develop BDD but also tend to have a rigid and perfectionist belief regarding how they should look, which further leads to negative self-evaluation and low self-esteem. Lastly, our study throws light on the fact that adolescents having poor economic status at home are more likely to develop BDD as compared to the ones with very good and excellent economic status in their families. Likewise, a study in Tamil Naidu, southern India [19], shows the increased prevalence of BDD in children and young adults of lower socioeconomic status, which may be explained by the fact that such adolescents try to fit into ideal standards set by the higher society and tend to seek approval from the world.

In the quest for acceptance, they tend to post more pictures and reels for which they try to maintain the perfect body to get more and more followers in order to get recognition and approval [19]. Not only in India, but this association between lower family income and BDD inclination has been studied globally. A study in Saudi Arabia [20] showed that in the struggle to fit among their peers of higher family income (having access to excellent facilities to maintain their bodies), adolescents of lower financial status tried to copy them and ultimately found themselves dissatisfied with their bodies. Hence, our research points out the rising body dysmorphia in adolescents in India due to the heavy use of social media and the various factors impacting it.

Limitations

While our study delivers a good understanding of how social media usage is linked to the development of BDD and the impact of gender and age on the prevalence of BDD, our study has certain limitations. Although the responses to the questionnaire were kept anonymous to avoid volunteer and observer bias and to increase confidentiality and privacy, some students might not disclose all the details due to the shame that still prevails in society regarding such disorders. Self-reported responses might affect the accuracy of responses. Lastly, our questionnaire was in simple language and without any leading questions, but there could be self-report bias since the participants were the only reporters for all study variables, which participants' opinions might influence, and this being a cross-sectional study might affect the accuracy of responses.

## Conclusions

Our study throws light on how excessive usage of social media platforms is associated with rising body dissatisfaction and dysmorphia in adolescents and the impact of gender, age, and financial status on it. The future version of this manuscript will also cover all adolescents (10-19 years of age) and add some more important strategies to overcome rising body dissatisfaction and dysmorphia. Social media filters have the potential to affect many adolescents’ self-esteem and their perceptions of their body image. Parents, teachers, doctors, counselors, and friends play a very important role in helping to overcome BDD by recognizing it in the early stages and providing emotional and professional support. Policymakers and governments should also intervene to regulate content guidelines of various platforms that promote fake ideal beauty standards and to improve the role of social media in society. Social media influencers instead of promoting beauty products should try to create awareness about the rising body dysmorphia and how to deal with the negative body image. With the right resources, we can help the younger generation to find beauty within themselves and not through these beauty filters. Emphasis should also be on educating adolescents through various campaigns that society’s standard of beauty does not define who they are.

## Appendices

Pre-validated questionnaire used in our study [8]

**Table 5:** Questionnaire (part 1)

Sociodemographic data							
Age (in years)	13 ( )	14 ( )	15 ( )		16 ( )		17 ( )
Gender	Male ( )			Female ( )			
Economic status (family monthly income in rupees)	Lower class (<2500 rs) ( )	Lower-middle (2500-5000 rs) ( )	Middle class (5000-10000 rs) ( )			Upper class (>10, 000 rs) ( )	

**Table 6:** Questionnaire (part 2)

Social media use practices				
Time spent on Instagram/Snapchat/Facebook etc. per day	<1 hour ( )	1-3 hours ( )	4-7 hours ( )	>7 hours ( )
Take selfies	Never ( )	Rarely ( )	Sometimes ( )	Always ( )
Use filters to edit selfies	Never ( )	Rarely ( )	Sometimes ( )	Always ( )
Share selfies with others	Never ( )	Rarely ( )	Sometimes ( )	Always ( )

**Table 7:** Questionnaire (part 3)

Have you ever	Never	Rarely	Sometimes	All of the time
Been very concerned about some aspect of your appearance?	0 ( )	1 ( )	2 ( )	3 ( )
Considered yourself misformed or misshapen in some way (e.g., nose/hair skin/sexual organs/overall body build)?	0 ( )	1 ( )	2 ( )	3 ( )
Considered your body to be malfunctional in some way (e.g., excessive body odour, flatulence, sweating)?	0 ( )	1 ( )	2 ( )	3 ( )
Consulted or felt you needed to consult a plastic surgeon/dermatologist/physician about these concerns?	0 ( )	1 ( )	2 ( )	3 ( )
Been told by others/doctor that you are normal in spite of you strongly believing that something is wrong with your appearance or bodily functioning?	0 ( )	1 ( )	2 ( )	3 ( )
Spent a lot of time worrying about a defect in your appearance/bodily functioning?	0 ( )	1 ( )	2 ( )	3 ( )
Spent a lot of time covering up defects in your appearance/bodily functioning?	0 ( )	1 ( )	2 ( )	3 ( )
